# Potential Adverse Events Reported With the Janus Kinase Inhibitors Approved for the Treatment of Rheumatoid Arthritis Using Spontaneous Reports and Online Patient Reviews

**DOI:** 10.3389/fphar.2021.792877

**Published:** 2022-01-11

**Authors:** Yun-Kyoung Song, Junu Song, Kyungim Kim, Jin-Won Kwon

**Affiliations:** ^1^ College of Pharmacy, Daegu Catholic University, Gyeongsan, South Korea; ^2^ Department of Computer Science, Viterbi School of Engineering, University of Southern California, Los Angeles, CA, United States; ^3^ College of Pharmacy, Korea University, Sejong, South Korea; ^4^ Institute of Pharmaceutical Science, Korea University, Sejong, South Korea; ^5^ BK21 FOUR Community-Based Intelligent Novel Drug Discovery Education Unit, College of Pharmacy and Research Institute of Pharmaceutical Sciences, Kyungpook National University, Daegu, South Korea

**Keywords:** Janus kinase inhibitors, rheumatoid arthritis, adverse event reporting systems, online patient reviews, potential adverse events

## Abstract

The aim of this study was to analyze the potential adverse events (AEs) caused by Janus kinase (JAK) inhibitors, including tofacitinib, baricitinib, and upadacitinib, used to treat rheumatoid arthritis using spontaneous AE reports from the FDA (FAERS) and interpreting them in correlation with those from Korea (KAERS) and an online patient review (WebMD). Potential AEs were identified based on a disproportionality analysis using the proportional reporting ratio (PRR), reporting odds ratio (ROR), and the information component (IC). A total of 23,720 reports were analyzed from FAERS database, of which 91.5% were reports on tofacitinib. Potentially important medical AEs related to infections were reported frequently, as well as thromboembolism-related AEs. The AEs, such as malignancy, interstitial lung diseases, myocardial infarction, and gastrointestinal disorder, also reported. In an online patient review report, the ineffectiveness of the drug and gastrointestinal AEs were frequently reported. Infection with baricitinib and symptoms related to pain or edema due to upadacitinib were the main discomfort experienced by patients. In conclusion, the results of this study highlight the possible safety issues associated with JAK inhibitors. Routine clinical observations and further research using various real-world databases are needed.

## Introduction

Janus kinase (JAK) inhibitors are small molecules that block the activity of one or more intracellular tyrosine kinases (JAK1, JAK2, JAK3, and tyrosine-protein kinase TYK2). Defects in JAKs cause severe immunosuppression in humans; thus, JAK inhibitors could be targets for immunosuppressive therapy (Winthrop, 2017). JAK-mediated signaling is involved in the pathogenesis of rheumatoid arthritis (RA). Tofacitinib is the first JAK inhibitor commercially approved for the treatment of RA, gaining approval by the United States Food and Drug Association (FDA) in 2012 and the Korean Ministry of Food and Drug Safety (MFDS) in 2014. Since then, baricitinib and upadacitinib were approved by the FDA in 2017 and 2019, respectively, and later started to be used in Korea to manage RA in patients with poor prognostic factors who fail initial treatment with conventional synthetic disease-modifying antirheumatic drugs (DMARDs) (United States Food and Drug Administration; Ministry of Food and Drug Safety, 2021; Angelini et al., 2020). However, increasing evidence suggests that JAK inhibitors may not be suitable for patients at risk for infection and thromboembolic events because the drugs block intracellular signaling pathways of inflammatory cytokines, which are relevant to host defense mechanisms, and may also affect thrombopoietin signaling and platelet homeostasis adversely (Bechman et al., 2019; Gadina et al., 2019; Harigai, 2019).

Adverse drug reactions are a significant cause of morbidity and mortality worldwide, and are responsible for approximately 6.5% of hospital admissions, making them crucial in healthcare decision-making (Lazarou et al., 1998; Pirmohamed et al., 2004). Moreover, it was reported that treatment discontinuation was associated with adverse events (AEs) of drugs used in patients with RA (Sakai et al., 2012). Spontaneous reporting data from the FDA’s Adverse Event Reporting System (FAERS) or the Korea Adverse Event Reporting System (KAERS) has been utilized for identifying the potential association between drugs and AEs and detecting rare events in post-marketing surveillance of drug safety due to the inherent limitations of clinical trials such as stringent trial design, strict enrollment criteria, relatively small sample size and limited follow-up duration (Sakaeda et al., 2013). However, due to a bias risk or under-reporting of the self-reported nature of the database, social media may serve as complementary sources of adverse drug reaction information from the patient perspective (Hazell and Shakir, 2006; Smith et al., 2018).

Various JAK inhibitors are associated with slightly different toxicity profiles, partially due to their different activities against JAK1−JAK3 (Scott et al., 2018; Harigai, 2019). However, the safety analysis of JAK inhibitors using these real-world data focused only on the risk of thrombotic events, and data are lacking regarding the comparative analysis of real-world safety of JAK inhibitors used for RA comprehensively (Verden et al., 2018; Peng et al., 2020). The present study aimed to analyze the potential AEs of JAK inhibitors used for RA, such as tofacitinib, baricitinib, and upadacitinib, using spontaneous AE reports from the FDA, and interpret them in correlation with spontaneous reports from Korea and online patient reviews.

## Materials and Methods

### Study Design and Ethical Issues

This was a retrospective pharmacovigilance disproportionality analysis using data from the FAERS, the KAERS and the WebMD. The study was approved by the institutional review board of Daegu Catholic University (IRB No. CUIRB-2020-E003) and waived the requirement for informed consent because all patient data were anonymized and de-identified prior to the retrospective analysis.

### Data Collection

Reported AE cases related to JAK inhibitors used for RA (tofacitinib, baricitinib, and upadacitinib) were analyzed using the FAERS, KAERS, and WebMD internet message boards. The FAERS and KAERS are databases that contain information on AE and medication error reports submitted to United States FDA or Korea Institute of Drug Safety and Risk Management spontaneously (U.S. Food and Drug Administration, 2020; Korea Institute of Drug Safety and Risk Management(KIDS), 2021). WebMD is an United States corporation and provides web-based health-related services. The Drugs and Supplements database in WebMD provides the overview information which includes ratings to three aspects of the medications such as effectiveness, ease of use and satisfaction, reviews and recommendations for medications that patients have tried (WebMD, 2021). The patient comments from WebMD were mainly analyzed in this study. The FAERS database was searched for reports on all FDA-approved JAK inhibitors in 2013–2020 (U.S. Food and Drug Administration, 2020). Records without notification or case number and the name of the suspected drug or the adverse reaction were excluded. Cases with patients aged less than 20 years and those aged over 100 years were excluded. Duplicate reports were deleted prior to the data analysis. The generic and brand names of all approved JAK inhibitors for RA, as well as their acronyms, were used in this study. Only initial reports where the role code was set to the primary suspect drug were selected. Cases marked as initial reports were selected. Adverse events were recorded using the preferred terms (PTs) from the Medical Dictionary for Regulatory Activities (MedDRA), and these PTs were categorized into their primary system organ classes (SOCs) in the MedDRA (Brown et al., 1999). Two or more PTs reported in one report were counted as different AEs. Serious AEs (SAEs) were classified as death, life-threatening, hospitalization, disability, or congenital anomaly, requiring intervention to prevent.

For the KAERS dataset, we analyzed the AEs reported by adults aged 20 years and older initially after taking JAK inhibitors in 2013–2019 (Korea Institute of Drug Safety and Risk Management). The following reports were excluded from analyses: reports on concomitant or interacting drugs, duplicate reports, and reports with unlikely or not-applicable causality based on the World Health Organization-Uppsala Monitoring Centre (WHO-UMC) criteria (The Uppsala Monitoring Centre, 2013). Since the KAERS uses the AE term based on WHO Adverse Reaction Terminology (WHO-ART), two researchers (Y.K.S. and K.K.) independently converted the term into a PT corresponding to MedDRA (Brown et al., 1999).

For text mining, the WebMD data for patient reviews recorded until December 31, 2020, after taking the JAK inhibitor, were downloaded using the Python-based library Beautiful Soup (WebMD, 2021). A total of 104, 5, and 21 reviews were collected for tofacitinib, baricitinib, and upadacitinib, respectively. Each reported symptom was manually assigned to a PT in the MedDRA terminology by two independent researchers (Y.K.S. and K.K.; Brown et al., 1999). Throughout the process, any disagreement was resolved either by discussion between the two researchers or by considering the opinion of an additional researcher (J.W.K.) to reach a consensus.

### Statistical Analysis

Disproportionality was analyzed using the proportional reporting ratio (PRR), reporting odds ratio (ROR), and information component (IC) to detect potential adverse events from the FAERS database. A two-by-two contingency table was composed of 1) the number of co-occurrences of interest (JAK inhibitor-specific AE), 2) the number of co-occurrences with the drug of interest, but without the AE of interest, 3) those without the drug of interest, but with the AE of interest, and 4) those without either, to detect spontaneous signals for a potentially increased risk of drug-related AEs. A potential AE of clinical relevance related to the use of each JAK inhibitor was defined when at least one of the three indices met the criteria described as follows (Sakaeda et al., 2013). Using the PRR, a potential AE was detected if the number of co-occurrences is three or more and the PRR is two or more, with an associated chi-square value with Yates’s correction of 4 or more (Evans et al., 2001). For the ROR, a potential AE was detected if the lower limit of the two-sided 95% confidence interval (95% CI) exceeds 1 (van Puijenbroek et al., 2002). Detection of the potential AE using the IC was done if the lower limit of the 95% CI value exceeds 0 (Bate et al., 1998). Underlying disease-related AEs such as rheumatoid arthritis were removed from the analysis (Hoffman et al., 2016). The Important Medical Event Terms (IME) list was used to identify the potentially important medical AEs based on their seriousness and clinical importance (European Medicines Agency, 2021c). We used Pandas, a Python software library for data manipulation, and SAS version 9.4 (SAS Institute, Cary, NC, United States) to compile and process the data.

## Results

### Characteristics of Spontaneous Reports for Janus Kinase Inhibitors From the FAERS

During the study period, a total of 10,883,085 reports were submitted to the FAERS. As shown in Table 1, 23,720 reports were adverse events in which JAK inhibitors were reported as the primary suspect agents. Of these, tofacitinib, baricitinib, and upadacitinib were reported in 91.5% (*n* = 21,708), 4.1% (*n* = 980), and 4.4% (*n* = 1,032), respectively. The mean age of patients with AEs related to JAK inhibitors was 59.9 years, and 54.3% (*n* = 12,890) of the patients were in their 50’s and 60’s. The AEs reported in women accounted for 80.3% (*n* = 19.045). Most AEs of tofacitinib (86.5%) were reported after 2015, and 56.6 and 88.9% of AEs of baricitinib and upadacitinib were reported within 2 years after 2019, respectively. Reports from healthcare professionals made up 21.2% of reports. Most reports came from North America (85.4%), followed by South America (5.2%). Reports from Asian countries accounted for only 2.9%. However, for baricitinib, only 59.7% of the unique reports were from North America, 30.7% from Europe, and 8.8% from Asia. In the reports, 85.7% listed some rheumatoid disease as the indication. For tofacitinib, a total of 8,163 SAEs (37.6%) were reported, and 11.9% were hospitalizations. Baricitinib and upadacitinib had a total of 614 and 731 SAEs, respectively, with majority of them being hospitalizations or others. Average 2.3 AEs were reported in each report.

**TABLE 1:** Demographic characteristics of population who reported adverse events of JAK inhibitors in the FAERS database.

	Tofacitinib	Baricitinib	Upadacitinib	Total
Number of reports	21,708	980	1,032	23,720
Age, years, mean (SD)	58.9 (12.7)	61.5 (12.7)	59.7 (12.0)	59.9 (12.1)
20’s−30’s, n (%)	1,505 (6.9)	47 (4.80)	43 (4.2)	1,595 (6.7)
40’s, n (%)	2,509 (11.6)	102 (10.4)	85 (8.2)	2,696 (11.4)
50’s, n (%)	5,838 (26.9)	176 (18.0)	218 (21.1)	6,232 (26.3)
60’s, n (%)	6,183 (28.5)	259 (26.4)	216 (20.9)	6,658 (28.1)
70’s, n (%)	3,091 (14.2)	182 (18.6)	119 (11.5)	3,392 (14.3)
80’s or older, n (%)	865 (4.0)	47 (4.8)	31 (3.0)	943 (4.0)
Unknown, n (%)	1,717 (7.9)	179 (18.3)	320 (31.0)	2,216 (9.3)
Gender, n (%)				
Male	4,121 (19.0)	187 (19.1)	189 (18.3)	4,497 (19.0)
Female	17,457 (80.4)	770 (78.6)	818 (79.3)	19,045 (80.3)
Unknown	130 (0.6)	23 (2.4)	25 (2.4)	178 (0.8)
Reporting year, n (%)				
2019–2020	7,484 (34.5)	555 (56.6)	917 (88.9)	8,956 (37.8)
2017–2018	7,231 (33.3)	395 (40.3)	19 (1.8)	7,645 (32.2)
2015–2016	4,084 (18.8)	2 (0.2)	1 (0.1)	4,087 (17.2)
2013–2014	2,210 (10.2)	2 (0.2)	0	2,212 (9.3)
Unknown	699 (3.2)	26 (2.7)	95 (9.2)	820 (3.5)
Reporter, n (%)				
Healthcare professionals	4,352 (20)	398 (40.1)	281 (27.2)	5,031 (21.2)
Consumers	119 (0.5)	3 (0.3)	8 (0.8)	130 (0.5)
Others	11(0.05)	1 (0.1)	0	12 (0.05)
Unknown	17,239 (79.4)	578(59.0)	743 (72.0)	18,560 (78.2)
Country, n (%)				
North America	18,722 (86.2)	585 (59.7)	948 (91.9)	20,255 (85.4)
South America	1,222 (5.6)	3 (0.3)	13 (1.3)	1,238 (5.2)
Europe	334 (1.5)	301 (30.7)	58 (5.6)	693 (2.9)
Asia	597 (2.8)	86 (8.8)	10 (1.0)	693 (2.9)
Others	140 (0.6)	5 (0.5)	3 (0.3)	148 (0.6)
Unknown	693 (3.2)	0	0	693 (2.9)
Indication for the use of JAK inhibitors, n (%)				
Rheumatoid disease	18,649 (85.9)	763 (77.9)	913 (88.5)	20,325 (85.7)
Crohn’s disease	61 (0.3)	0	3 (0.3)	64 (0.3)
Psoriasis	125 (0.6)	3 (0.3)	1 (0.1)	129 (0.5)
Others or unknown	2,873 (13.2)	214 (21.8)	115 (11.1)	3,202 (13.5)
Serious adverse events				
Death	514 (2.4)	43 (4.4)	29 (2.8)	586 (2.5)
Life-threatening	145 (0.7)	47 (4.8)	9 (0.9)	201 (0.8)
Hospitalization	2,580 (11.9)	281 (28.7)	259 (25.1)	3,120 (13.2)
Disability	111 (0.5)	18 (1.8)	5 (0.5)	134 (0.6)
Congenital anomaly	10 (<0.1)	2 (0.2)	0	12 (0.1)
Others	4,803 (22.1)	223 (22.8)	429 (41.6)	5,455 (23.0)
Number of adverse events per reports, mean (SD)	2.3 (2.0)	2.1 (1.7)	2.4 (2.2)	2.3 (2.0)

### Potential Adverse Events of Janus Kinase Inhibitors Used for Rheumatoid Arthritis From the FAERS Database

Table 2 presents the distribution of SOCs for a total of 32,760, 1,071, and 1,381 potential AEs of tofacitinib, baricitinib, and upadacitinib, respectively. As SOC terms, general disorders were highest in tofacitinib (29.0%), and infections were highest in both baricitinib and upadacitinib (31.7 and 23.5%, respectively). For tofacitinib, the examples of potential AEs in the general disorders were drug ineffective (2,176/9,499, 22.9%) and condition aggravated (1,373/9,499, 14.5%; data not shown). In patients taking baricitinib, the main potential AEs among infection SOCs were general infections (24/339, 7.1%) and herpes zoster (23/339, 6.8%, data not shown). In contrast, in patients with upadacitinib, the main potential AEs as infections were urinary tract infection (38/325, 11.7%) and pneumonia (35/325, 10.8%, data not shown).

**TABLE 2:** Distribution of System Organ Classes (SOCs) for potential adverse events of JAK inhibitors used for rheumatoid arthritis from the FAERS database.

SOCs	Tofacitinib	Baricitinib	Upadacitinib	Total
General disorders and administration site conditions	9,499 (29.0)	69 (6.4)	158 (11.4)	9,726 (27.6)
Infections and infestations	5,487 (16.8)	339 (31.7)	325 (23.5)	6,151 (17.5)
Musculoskeletal and connective tissue disorders	3,756 (11.5)	77 (7.2)	177 (12.8)	4,010 (11.4)
Injury, poisoning and procedural complications	2,590 (7.9)	47 (4.4)	125 (9.1)	2,762 (7.8)
Gastrointestinal disorders	2,338 (7.1)	64 (6.0)	35 (2.5)	2,437 (6.9)
Respiratory, thoracic and mediastinal disorders	1,913 (5.8)	80 (7.5)	42 (3.0)	2,035 (5.8)
Nervous system disorders	1,669 (5.1)	30 (2.8)	42 (3.0)	1,741 (4.9)
Investigations	1,391 (4.3)	49 (4.6)	65 (4.7)	1,505 (4.3)
Surgical and medical procedures	1,060 (3.2)	107 (10.0)	252 (18.3)	1,419 (4.0)
Psychiatric disorders	728 (2.2)	1 (0.1)	6 (0.4)	735 (2.1)
Skin and subcutaneous tissue disorders	434 (1.3)	17 (1.6)	3 (0.2)	454 (1.3)
Neoplasms benign, malignant and unspecified	334 (1.0)	56 (5.2)	37 (2.7)	427 (1.2)
Renal and urinary disorders	349 (1.1)	24 (2.2)	11 (0.8)	384 (1.1)
Vascular disorders	312 (1.0)	39 (3.6)	29 (2.1)	380 (1.1)
Immune system disorders	198 (0.6)	2 (0.2)	8 (0.6)	208 (0.6)
Social circumstances	146 (0.5)	6 (0.6)	0	152 (0.4)
Hepatobiliary disorders	134 (0.4)	6 (0.6)	5 (0.4)	145 (0.4)
Metabolism and nutrition disorders	122 (0.4)	2 (0.2)	6 (0.4)	130 (0.4)
Eye disorders	101 (0.3)	5 (0.5)	22 (1.6)	128 (0.4)
Ear and labyrinth disorders	103 (0.3)	3 (0.3)	3 (0.2)	109 (0.3)
Blood and lymphatic system disorders	28 (0.1)	28 (2.6)	1 (0.1)	57 (0.2)
Reproductive system and breast disorders	31 (0.1)	6 (0.6)	8 (0.6)	45 (0.1)
Cardiac disorders	8 (<0.1)	8 (0.8)	20 (1.5)	36 (0.1)
Endocrine disorders	20 (<0.1)	1 (0.1)	0	21 (0.1)
Product issues	4 (<0.1)	2 (0.2)	0	6 (<0.1)
Congenital, familial and genetic disorders	4 (<0.1)	1 (0.1)	1 (0.1)	6 (<0.1)
Pregnancy, puerperium and perinatal conditions	1 (<0.1)	2 (0.2)	0	3 (<0.1)
Total	32,760 (100.0)	1,071 (100.0)	1,381 (100.0)	35,212 (100.0)

The top 10 potential IMEs from FARES and the listing status of Korean labels are presented in Table 3. Tofacitinib, baricitinib, and upadacitinib were associated with infections (e.g., pneumonia), vascular disorders (e.g., thrombosis or deep vein thrombosis), and renal and urinary disorders (e.g., renal impairment). Cataract surgery was specified for tofacitinib and baricitinib, and breast cancer was indicated in baricitinib and upadacitinib. Hematochezia for tofacitinib and respiratory disease (e.g., pulmonary embolism, interstitial lung disease, respiratory failure) for baricitinib were reported. Nerve system disorders (e.g., loss of confusion), cardiac disorders (e.g., myocardial infarction), and systematic lupus syndrome for upadacitinib were also reported. Some of them were not listed on the Korean label.

**TABLE 3:** Top 10 potentially important medical adverse events (IME) of JAK inhibitors used for rheumatoid arthritis from FAERS database.

Drugs	SOC	IME	Primary suspected cases, n	PRR (χ^2^)	ROR (95% CI)	IC (lower limit of 95% CI)	Korean drug label^#^
Tofacitinib	Infections and infestations	Pneumonia	478	1.91 (205.72)	1.92 (1.75–2.10)^*^	0.93 (0.78)^*^	O
	Vascular disorders	Thrombosis	98	1.51 (16.44)	1.51 (1.24–1.85)^*^	0.59 (0.26)^*^	O
	Gastrointestinal disorders	Haematochezia	97	2.44 (80.23)^*^	2.44 (2.00–2.98)^*^	1.27 (0.93)^*^	X
	Renal and urinary disorders	Nephrolithiasis	96	2.85 (112.61)^*^	2.85 (2.33–3.49)^*^	1.49 (1.15)^*^	X
	Metabolism and nutrition disorders	Diabetes mellitus	95	1.80 (32.93)	1.80 (1.47–2.21)^*^	0.84 (0.50)^*^	X
	Eye disorders	Cataract	84	1.88 (33.52)	1.88 (1.52–2.33)^*^	0.90 (0.54)^*^	X
	Renal and urinary disorders	Renal impairment	80	1.39 (8.35)	1.39 (1.12–1.73)^*^	0.47 (0.10)^*^	X
	Infections and infestations	Diverticulitis	72	3.28 (110.82)^*^	3.28 (2.60–4.14)^*^	1.68 (1.29)^*^	O
	Infections and infestations	Kidney infection	70	4.52 (185.87)^*^	4.52 (3.57–5.73)^*^	2.13 (1.73)^*^	O
	Infections and infestations	Cellulitis	66	1.64 (15.91)	1.64 (1.29–2.09)^*^	0.71 (0.30)^*^	O
Baricitinib	Respiratory, thoracic and mediastinal disorders	Pulmonary embolism	27	9.62 (200.00)^*^	9.62 (6.66–14.23)^*^	3.05 (2.41)^*^	O
	Vascular disorders	Deep vein thrombosis	20	9.90 (151.12)^*^	9.90 (6.42–15.51)^*^	3.02 (2.27)^*^	O
	Infections and infestations	Pneumonia	17	1.62 (3.42)	1.62 (1.01–2.61)^*^	0.67 (-0.15)	O
	Infections and infestations	Cellulitis	14	8.31 (82.88)^*^	8.31 (4.94–14.15)^*^	2.73 (1.83)^*^	O
	Infections and infestations	Sepsis	11	3.31 (15.51)^*^	3.31 (1.84–6.01)^*^	1.59 (0.56)^*^	X
	Respiratory, thoracic and mediastinal disorders	Interstitial lung disease	8	6.01 (28.61)^*^	6.01 (3.01–12.08)^*^	2.21 (1.00)^*^	X
	Respiratory, thoracic and mediastinal disorders	Respiratory failure	8	4.00 (15.11)^*^	4.00 (2.00–8.03)^*^	1.76 (0.55)^*^	X
	Renal and urinary disorders	Renal impairment	7	2.90 (6.93)^*^	2.90 (1.38–6.11)^*^	1.36 (0.06)^*^	X
	Neoplasms benign, malignant and unspecified	Breast cancer	6	4.54 (13.20)^*^	4.54 (2.04–10.14)^*^	1.83 (0.42)^*^	X
	Infections and infestations	Diverticulitis	6	6.49 (22.62)^*^	6.49 (2.92–14.50)^*^	2.19 (0.77)^*^	X
Upadacitinib	Infections and infestations	Pneumonia	35	2.79 (38.57)^*^	2.81 (2.02–3.93)^*^	1.44 (0.88)^*^	O
	Nervous system disorders	Loss of consciousness	17	3.63 (29.88)^*^	3.65 (2.26–5.88)^*^	1.76 (0.94)^*^	X
	Cardiac disorders	Myocardial infarction	14	1.97 (5.75)	1.97 (1.17–3.34)^*^	0.93 (0.03)^*^	X
	Eye disorders	Cataract	13	5.81 (47.04)^*^	5.83 (3.38–10.06)^*^	2.30 (1.36)^*^	X
	Vascular disorders	Thrombosis	13	4.01 (26.44)^*^	4.02 (2.33–6.94)^*^	1.85 (0.91)^*^	O
	Renal and urinary disorders	Nephrolithiasis	9	5.32 (27.40)^*^	5.34 (2.77–10.27)^*^	2.12 (0.98)^*^	X
	Respiratory, thoracic and mediastinal disorders	Pulmonary embolism	8	2.39 (5.13)^*^	2.39 (1.19–4.79)^*^	1.14 (-0.07)	O
	Vascular disorders	Deep vein thrombosis	7	2.90 (6.92)^*^	2.91 (1.38–6.10)^*^	1.36 (0.06)^*^	O
	Musculoskeletal and connective tissue disorders	Systemic lupus erythematosus	7	6.20 (25.55)^*^	6.22 (2.96–13.06)^*^	2.20 (0.90)^*^	X
	Neoplasms benign, malignant and unspecified	Breast cancer	6	3.80 (9.75)^*^	3.81 (1.71–8.49)^*^	1.64 (0.23)^*^	X

*Statistically significant association, i.e., the adverse events are detected as signals.

^#^“O” means that the IME, is described in the Korean drug label, and “X” means not listed.

### Frequently Reported Adverse Events of Janus Kinase Inhibitors Used for Rheumatoid Arthritis From Spontaneous Reports and Online Patient Reviews

**FIGURE 1:**
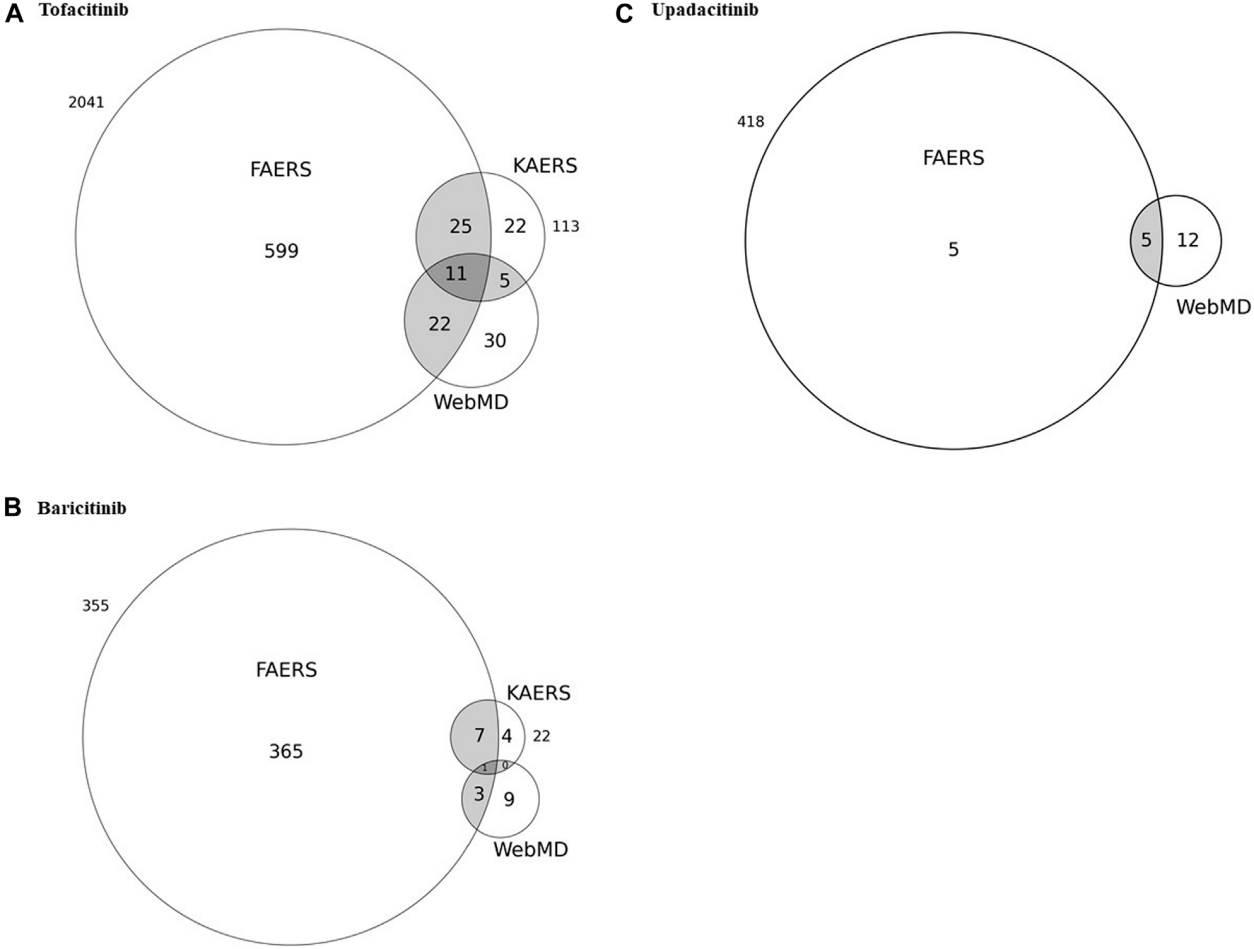
Venn diagrams of potential adverse events detected from the United States FDA adverse event reporting system (FAERS) in correlation with the spontaneous reports from Korea as well as adverse events reported through patient reviews in WebMD for **(A)** tofacitinib, **(B)** baricitinib, and **(C)** upadacitinib.

Figure 1 and Table 4 show the potential AEs detected from FAERS and KAERS, as well as AEs reported in the patient review in WebMD. For tofacitinib, 11 potential AEs such as drug ineffectiveness, headache, diarrhea, arthralgia, and pain in extremities were reported from all spontaneous reports and online patient reviews. The 25 potential AEs were detected only from spontaneous reports, among which pneumonia and fracture were reported relatively often in Korea. Twenty-two potential AEs were reported from FAERS and WebMD, and among 104 reviews in WebMD, fatigue (15) and weight increase (23) were the frequently reported potential AEs in patients taking tofacitinib. Although AEs such as nausea, dizziness, insomnia, constipation, and pruritus were reported from KAERS and WebMD, they were not included in the potential AEs due to disproportionality analysis using the FAERS database. In patients taking baricitinib, only urinary tract infection was the potential AE co-reported from FAERS, KAERS, and WebMD. From the spontaneous reports, seven potential AEs, such as pulmonary embolism, nasopharyngitis, and sepsis, were problems after taking the drug. Sinusitis, abdominal pain, and constipation were potential AEs reported in the online patient review. Among the potential AEs of upadacitinib, pain, peripheral and joint swelling, contusion, and gastrointestinal edema have also been reported from WebMD. There were no data on upadacitinib from KAERS during the period of 2013–2019 since the drug was approved in June 2020 in Korea (Ministry of Food and Drug Safety).

**TABLE 4:** Potential adverse events detected from FAERS in correlation with the spontaneous reports from KAERS and adverse events reported in patient review in WebMD.

Classifi-cation	FAERS						WebMD	KAERS
	System organ class	Adverse event	Primary suspected cases, n	PRR (χ^2^)	ROR (95% CI)	IC (lower limit of 95% CI)	Cases, n	Suspected cases, n
Tofacitinib								
FAERS-KAERS-WebMD	General disorders and administration site conditions	Drug ineffective	2,176	1.68 (605.93)	1.71 (1.64–1.78)^*^	0.74 (0.67)^*^	8	3
	Nervous system disorders	Headache	1,212	2.11 (709.74)^*^	2.14 (2.02–2.26)^*^	1.07 (0.98)^*^	8	1
	Gastrointestinal disorders	Diarrhoea	830	1.48 (129.18)	1.49 (1.39–1.59)^*^	0.56 (0.45)^*^	5	1
	Musculoskeletal and connective tissue disorders	Arthralgia	727	2.16 (451.47)^*^	2.18 (2.02–2.34)^*^	1.10 (0.98)^*^	3	3
	Musculoskeletal and connective tissue disorders	Pain in extremity	574	2.24 (392.21)^*^	2.25 (2.08–2.45)^*^	1.16 (1.02)^*^	2	1
	Respiratory, thoracic and mediastinal disorders	Cough	475	2.14 (286.03)^*^	2.15 (1.96–2.35)^*^	1.09 (0.94)^*^	1	2
	Infections and infestations	Sinusitis	437	4.96 (1,361.83)^*^	5.00 (4.55–5.50)^*^	2.29 (2.13)^*^	1	1
	Gastrointestinal disorders	Abdominal pain upper	372	2.07 (204.08)^*^	2.08 (1.88–2.30)^*^	1.04 (0.87)^*^	11	2
	Infections and infestations	Herpes zoster	350	7.23 (1,837.09)^*^	7.28 (6.54–8.09)^*^	2.82 (2.64)^*^	1	6
	General disorders and administration site conditions	Pyrexia	341	1.32 (25.77)	1.32 (1.19–1.47)^*^	0.40 (0.22)^*^	1	3
	Musculoskeletal and connective tissue disorders	Back pain	262	1.34 (22.17)	1.34 (1.19–1.51)^*^	0.42 (0.21)^*^	2	5
FAERS-KAERS	General disorders and administration site conditions	Condition aggravated	1,373	6.35 (6,098.89)^*^	6.50 (6.16–6.86)^*^	2.64 (2.55)^*^	NA	1
	Infections and infestations	Pneumonia	478	1.91 (205.72)	1.92 (1.75–2.10)^*^	0.93 (0.78)^*^	NA	7
	Infections and infestations	Infection	366	3.16 (533.68)^*^	3.17 (2.86–3.52)^*^	1.65 (1.47)^*^	NA	3
	Vascular disorders	Hypertension	185	1.19 (5.32)	1.19 (1.03–1.37)^*^	0.25 (<0.01)^*^	NA	1
	Investigations	Hepatic enzyme increased	148	3.04 (198.81)^*^	3.04 (2.59–3.58)^*^	1.59 (1.31)^*^	NA	1
	Musculoskeletal and connective tissue disorders	Arthropathy	121	2.80 (137.14)^*^	2.80 (2.34–3.35)^*^	1.47 (1.17)^*^	NA	2
	Infections and infestations	Cystitis	103	3.95 (221.26)^*^	3.95 (3.25–4.80)^*^	1.95 (1.62)^*^	NA	1
	Renal and urinary disorders	Renal impairment	80	1.39 (8.35)	1.39 (1.12–1.73)^*^	0.47 (0.10)^*^	NA	1
	Respiratory, thoracic and mediastinal disorders	Productive cough	72	2.06 (37.77)^*^	2.06 (1.63–2.59)^*^	1.02 (0.63)^*^	NA	1
	Infections and infestations	Cellulitis	66	1.64 (15.91)	1.64 (1.29–2.09)^*^	0.71 (0.30)^*^	NA	2
	Neoplasms benign, malignant and unspecified (incl cysts and polyps)	Neoplasm malignant	59	1.36 (5.31)	1.36 (1.06–1.76)^*^	0.44 (0.01)^*^	NA	2
	Respiratory, thoracic and mediastinal disorders	Respiratory disorder	50	2.16 (29.77)^*^	2.16 (1.64–2.85)^*^	1.09 (0.62)^*^	NA	1
	Infections and infestations	Staphylococcal infection	40	1.61 (8.56)	1.61 (1.18–2.19)^*^	0.67 (0.15)^*^	NA	2
	Neoplasms benign, malignant and unspecified (incl cysts and polyps)	Lung neoplasm malignant	38	1.91 (15.39)	1.91 (1.39–2.62)^*^	0.91 (0.37)^*^	NA	1
	Blood and lymphatic system disorders	Lymphadeno-pathy	35	1.36 (2.91)	1.36 (0.97–1.89)	0.43 (-0.13)	NA	1
	Investigations	Red blood cell sedimentation rate increased	32	4.32 (77.44)^*^	4.32 (3.05–6.13)^*^	2.03 (1.44)^*^	NA	1
	Injury, poisoning and procedural complications	Fracture	29	1.49 (4.15)	1.49 (1.03–2.14)^*^	0.56 (−0.06)	NA	5
	Infections and infestations	Bacterial infection	28	2.28 (18.68)^*^	2.28 (1.57–3.30)^*^	1.15 (0.52)^*^	NA	1
	Infections and infestations	Arthritis infective	21	6.08 (82.73)^*^	6.09 (3.95–9.37)^*^	2.43 (1.69)^*^	NA	1
	Neoplasms benign, malignant and unspecified (incl cysts and polyps)	Lymphoma	20	1.76 (5.79)	1.76 (1.13–2.73)^*^	0.79 (0.04)^*^	NA	1
	Infections and infestations	Pharyngitis	17	1.72 (4.43)	1.72 (1.07–2.77)^*^	0.75 (−0.06)	NA	2
	Musculoskeletal and connective tissue disorders	Sjogren’s syndrome	14	3.59 (23.44)^*^	3.59 (2.12–6.09)^*^	1.71 (0.81)^*^	NA	1
	Infections and infestations	Appendicitis	14	2.28 (8.81)^*^	2.29 (1.35–3.87)^*^	1.12 (0.22)^*^	NA	1
	Infections and infestations	Pneumococcal sepsis	4	10.70 (25.33)^*^	10.70 (3.95–28.93)^*^	2.35 (0.58)^*^	NA	1
	Neoplasms benign, malignant and unspecified (incl cysts and polyps)	Carcinoma *in situ*	1	8.08 (1.11)	8.08 (1.11–58.71)^*^	1.26 (−2.52)	NA	1
FAERS-WebMD	General disorders and administration site conditions	Fatigue	820	1.22 (33.12)	1.23 (1.14–1.31)^*^	0.29 (0.17)^*^	15	NA
	Infections and infestations	Urinary tract infection	446	3.54 (803.04)^*^	3.56 (3.24–3.91)^*^	1.81 (1.65)^*^	1	NA
	Infections and infestations	Influenza	383	3.91 (819.22)^*^	3.93 (3.56–4.35)^*^	1.95 (1.78)^*^	2	NA
	Respiratory, thoracic and mediastinal disorders	Oropharyngeal pain	339	4.18 (809.58)^*^	4.21 (3.78–4.68)^*^	2.04 (1.87)^*^	6	NA
	Investigations	Weight increased	331	1.88 (134.41)	1.88 (1.69–2.10)^*^	0.90 (0.72)^*^	23	NA
	Investigations	Blood cholesterol increased	303	11.30 (2,747.27)^*^	11.37 (10.13–12.75)^*^	3.43 (3.24)^*^	7	NA
	Gastrointestinal disorders	Abdominal discomfort	290	1.95 (133.17)	1.96 (1.74–2.20)^*^	0.96 (0.76)^*^	2	NA
	Psychiatric disorders	Depression	262	1.41 (30.19)	1.41 (1.25–1.59)^*^	0.49 (0.28)^*^	7	NA
	Infections and infestations	Bronchitis	260	3.88 (547.10)^*^	3.89 (3.44–4.40)^*^	1.94 (1.73)^*^	2	NA
	Respiratory, thoracic and mediastinal disorders	Nasal congestion	242	5.37 (842.88)^*^	5.39 (4.74–6.12)^*^	2.39 (2.18)^*^	3	NA
	Skin and subcutaneous tissue disorders	Alopecia	231	1.18 (6.17)	1.18 (1.04–1.34)^*^	0.24 (0.02)^*^	2	NA
	Investigations	Blood pressure increased	194	1.64 (47.79)	1.64 (1.43–1.89)^*^	0.71 (0.47)^*^	2	NA
	Gastrointestinal disorders	Dyspepsia	141	1.82 (51.36)	1.83 (1.55–2.15)^*^	0.86 (0.58)^*^	1	NA
	Gastrointestinal disorders	Stomatitis	88	1.89 (35.66)	1.89 (1.53–2.33)^*^	0.91 (0.55)^*^	1	NA
	Investigations	White blood cell count decreased	84	1.14 (1.34)	1.14 (0.92–1.42)	0.19 (−0.17)	1	NA
	Immune system disorders	Immune system disorder	73	6.91 (355.86)^*^	6.92 (5.48–8.72)^*^	2.71 (2.32)^*^	2	NA
	Infections and infestations	Respiratory tract infection	49	2.33 (35.58)^*^	2.33 (1.76–3.09)^*^	1.19 (0.72)^*^	2	NA
	Respiratory, thoracic and mediastinal disorders	Sinus congestion	41	3.51 (70.51)^*^	3.51 (2.58–4.78)^*^	1.76 (1.24)^*^	1	NA
	General disorders and administration site conditions	Hunger	16	1.72 (4.15)	1.72 (1.06–2.82)^*^	0.75 (−0.09)	1	NA
	Musculoskeletal and connective tissue disorders	Trigger finger	14	4.81 (38.04)^*^	4.81 (2.84–8.16)^*^	2.08 (1.17)^*^	1	NA
	General disorders and administration site conditions	Symptom recurrence	4	2.46 (2.14)	2.46 (0.92–6.58)	1.08 (−0.69)	7	NA
	Respiratory, thoracic and mediastinal disorders	Throat lesion	2	4.00 (1.97)	4.00 (0.99–16.10)	1.31 (−1.28)	1	NA
WebMD-KAERS	Gastrointestinal disorders	Nausea	636	0.91 (<0.01)	0.91 (0.84–0.99)	−0.13 (−0.26)	4	1
	Nervous system disorders	Dizziness	341	0.78 (<0.01)	0.78 (0.70–0.87)	−0.35 (−0.53)	3	3
	Psychiatric disorders	Insomnia	197	0.82 (<0.01)	0.82 (0.71–0.94)	−0.29 (−0.53)	2	1
	Gastrointestinal disorders	Constipation	184	1.06 (0.66)	1.06 (0.92–1.23)	0.09 (−0.15)	2	2
	Skin and subcutaneous tissue disorders	Pruritus	155	0.51 (<0.01)	0.51 (0.43–0.59)	−0.97 (−1.23)	3	2
Baricitinib								
FAERS-KAERS-WebMD	Infections and infestations	Urinary tract infection	20	3.76 (37.90)^*^	3.79 (2.44–5.88^*^	1.82 (1.07)^*^	1	1
FAERS-KAERS	Respiratory, thoracic and mediastinal disorders	Pulmonary embolism	27	9.62 (200.00)^*^	9.73 (6.66–14.23)^*^	3.05 (2.41)^*^	NA	1
	Infections and infestations	Nasopharyngitis	19	2.73 (19.24)^*^	2.75 (1.75–4.32)^*^	1.39 (0.62)^*^	NA	1
	Infections and infestations	Sepsis	11	3.31 (15.51)^*^	3.31 (1.84–6.01)^*^	1.59 (0.56)^*^	NA	1
	Cardiac disorders	Pericarditis	3	10.86 (17.88)^*^	10.87 (3.50–33.77)^*^	2.17 (0.10)^*^	NA	1
	Gastrointestinal disorders	Gastric ulcer perforation	2	57.00 (60.74)	57.05 (14.19–229.35)^*^	2.22 (−0.37)	NA	1
	Respiratory, thoracic and mediastinal disorders	Pulmonary infarction	2	44.98 (47.39)	45.02 (11.21–180.82)^*^	2.20 (−0.39)	NA	1
	Infections and infestations	Pyelonephritis	2	8.73 (7.04)	8.73 (2.18–34.97)^*^	1.78 (−0.82)	NA	1
FAERS- WebMD	Infections and infestations	Sinusitis	23	6.17 (94.62)^*^	6.23 (4.12–9.39)^*^	2.47 (1.78)^*^	1	NA
	Gastrointestinal disorders	Abdominal pain upper	17	2.25 (10.64)^*^	2.26 (1.40–3.65)^*^	1.12 (0.30)^*^	1	NA
	Gastrointestinal disorders	Constipation	14	1.93 (5.43)	1.94 (1.15–3.28)^*^	0.91 (<0.01)^*^	1	NA
Upadacitinib								
FAERS- WebMD	General disorders and administration site conditions	Pain	57	2.51 (50.53)^*^	2.54 (1.95–3.30)^*^	1.31 (0.87)	2	NA
	General disorders and administration site conditions	Peripheral swelling	21	2.91 (24.53)^*^	2.93 (1.91–4.50)^*^	1.48 (0.75)	1	NA
	Musculoskeletal and connective tissue disorders	Joint swelling	14	2.85 (15.02)^*^	2.86 (1.69–4.84)^*^	1.42 (0.52)	1	NA
	Injury, poisoning and procedural complications	Contusion	9	2.28 (5.28)^*^	2.29 (1.19–4.41)^*^	1.10 (−0.04)	1	NA
	Gastrointestinal disorders	Gastrointestinal oedema	1	15.36 (2.90)	15.36 (2.16–109.35)^*^	1.41 (−2.38)	1	NA

NA, not applicable.

*Statistically significant association, i.e., the adverse events are detected as signals.

## Discussion

As a novel targeted therapy, JAK inhibitors have enabled the treatment of RA to enter a new stage. Real-world evidence remains to be established to bridge the gap between randomized controlled trials and rheumatology clinics (Angelini et al., 2020). Therefore, it is necessary to generate a comprehensive analysis of potential AEs using various real-world databases. To the best of our knowledge, this is the first study that comprehensively compared the clinically relevant potential AEs for each JAK inhibitor using the FDA and Korean spontaneous reporting data, and analyzed them in association with open-ended patients’ opinions on discomfort after taking the medication. Various sources of AEs can exert different effects, each with unique advantages. Although voluntary spontaneous reports such as FAERS or KAERS may not capture accurate incidence information due to under- or over-reporting of known AEs and patients’ perspectives may be filtered through healthcare professionals and regulatory agencies, they contain millions of AE-related records. Online patient reviews in social media sites can be a direct source of pharmacovigilance from patient-generated experiences (Pierce et al., 2017; Li et al., 2020). It was reported that 59% of adults in the United States had looked online for health information, and a high volume of discussions about medical products is occurring online to share up-to-date concerns and reactions to medications publicly (Fox and Duggan, 2013). However, the patient-generated content of social media may have issues with the credibility, frequency and importance of the data, although WebMD is the specialized health-centered social networking sites (Sarker et al., 2015). Therefore, more potential novel adverse drug events could be detected by combining spontaneous reports and online patient reviews.

Tofacitinib was used for a relatively longer period compared to baricitinib and upadacitinib in patients with RA, so the number of reports on tofacitinib made up 91.6% of the total number of AE reports of JAK inhibitors (U.S. Food and Drug Administration and DrugsFDA, 2021). In the European database of suspected adverse drug reaction reports called EudraVigilance, reports on tofacitinib accounted for 89.0% (34,645/38,938) of the total number of reports on JAK inhibitors (European Medicines Agency, 2021a). It was reported that approximately 45% of patients diagnosed with RA were over 65 years of age, and female patients accounted for approximately 74% in the United States (Hunter et al., 2017). In this study, approximately 46.3% of the reports on JAK inhibitors were at least 60 years old, and reports from female patients accounted for 80.3%. Among the spontaneous AE reports, 86.2% of tofacitinib and 91.9% of upadacitinib were reported from North America, whereas only 59.7% of baricitinib were from North America, and 30.7% from Europe. It might be thought that baricitinib was approved by the European Medicines Agency in February 2017, so more AEs were reported in Europe, where it was used for about a year longer (European Medicines Agency, 2021b).

There are common safety concerns such as infections, thromboembolism and malignancy with JAK inhibitors. These drugs block intracellular signaling pathways of inflammatory cytokines relevant to the host defense mechanisms. The process of thrombopoietin signaling and cancer immunoediting might rely upon a variety of cytokines such as interferon gamma and cell types such as NK cell, which could be affected by JAK inhibition (Winthrop, 2017; Harigai, 2019). However, each JAK inhibitor currently approved for RA has different activities against JAK1−3 as follows: tofacitinib is preferentially a JAK 1 and 3 inhibitor, baricitinib is primarily a JAK 1 and 2 inhibitor, while upadacitinib selectively inhibits JAK1 which could potentially reduce JAK2 and JAK3-related side effects (Winthrop, 2017). Therefore, it might be thought that there were differences in AE parameters among the JAK inhibitors (Morinobu, 2020).

The most common and significant SOCs of AEs were infections, which corresponded to previous safety data (Winthrop, 2017; Bechman et al., 2019). Among the related IMEs, sepsis and diverticulitis were reported frequently, which were also found in the previous study but was not included in the Korean drug label (Peng et al., 2020). Sepsis was also reported in Koreans taking baricitinib, so further studies are needed to clarify the causal relationship between this AE and the drug. In addition, respiratory tract infections such as sinusitis, nasopharyngitis, influenza, and other infections such as herpes zoster, cellulitis, and urinary tract infections were the main potential AEs which patients had felt uncomfortable while taking tofacitinib or baricitinib. It was reported that whereas most infections associated with JAK inhibitors were bacterial, which was similar to that associated with biological therapy, a different risk profile related to viral infections has emerged on JAK inhibitors (Winthrop, 2017). Hence, it is necessary to monitor and educate patients about the symptoms associated with infections.

There is a growing concern that patients treated with JAK inhibitors may experience an increased risk of thromboembolism, which has led the FDA and Korean MFDS to require a warning for most JAK inhibitors currently on the market (United States Food and Drug Administration; Ministry of Food and Drug Safety; Winthrop, 2017). The findings from the current analysis, which includes a large population of patients treated with JAK inhibitors in a real-world setting, further support this observation. Among the top 10 potential IMEs, thrombosis, pulmonary embolism, and deep vein thrombosis were the main potential AEs occurring with tofacitinib or baricitinib. The ROR of pulmonary embolism was disproportionately high with baricitinib, as was the risk of deep vein thrombosis. It was also found to be a suspected case from the KAERS database. The potential AEs such as respiratory disorder or pulmonary infarction co-reported from both spontaneous reporting databases might be related to pulmonary embolism (Di Nisio et al., 2016). Our results are in close agreement with a previous study in which TEs were found in patients treated with JAK inhibitors with significant reporting rates (Setyawan et al., 2021). However, it is notable that there was an increased rate of thrombosis, pulmonary embolism, and deep vein thrombosis with upadacitinib used for about a year after approval, since in the previous study, FAERS data reported for approximately 45 days after upadacitinib approval were analyzed, and only pulmonary embolism was found to be a significant signal (Setyawan et al., 2021). However, this study, which analyzed the data over a period of 1.5 years, found more thromboembolism-related potential adverse events with upadacitinib, suggesting that further caution and analysis are needed in the future. Various JAK inhibitors are associated with slightly different toxicity profiles, and growing evidence, including this study, suggests that JAK inhibitors may not be suitable for patients at risk for infection and thromboembolic events.

Although JAK inhibitors may increase the risk of thromboembolism events, a recent meta-analysis of randomized clinical trials (RCTs) reported that JAK inhibitors did not significantly change cardiovascular risk, and there was no difference among JAK inhibitors for the occurrence of cardiovascular or thromboembolic events (Xie et al., 2019; Alves et al., 2021). However, caution is needed in its interpretation because of the limited follow-up period of the RCTs. In this study, the incidence of cardiac disorder-related potential AEs was relatively low (0.1%) in patients taking JAK inhibitors. However, myocardial infarction was the frequently reported potential IME in patients with upadacitinib, and pericarditis has been shown to be a potential AE for baricitinib in both the United States and Korea. Therefore, continuous post-marketing monitoring is necessary to analyze the correlation between JAK inhibitors and cardiovascular outcomes in patients with RA.

JAK inhibitors have been hypothesized to promote malignancy, which has not been identified in clinical trials, through the process of cancer immunoediting (Winthrop, 2017; Harigai, 2019). The most common malignancies related to exposure to tofacitinib were lung cancer, breast cancer, and lymphoma. The incidence rates of overall malignancy were 0.85 and 0.8 per 100 patient-years for tofacitinib and baricitinib, respectively (Curtis et al., 2016; Harigai, 2019). In this study, breast cancer was also reported as an IME in patients taking baricitinib with an ROR of 4.54 (95% CI, 2.04–10.14) and upadacitinib with an ROR of 3.81 (95% CI, 1.71–8.49). In addition, malignant neoplasms, malignant lung neoplasms, and lymphomas have also been reported from KAERS. The process by which the immune system destroys cancer cells relies on various cytokines and NK cells and could be affected by JAK inhibition. However, it was reported that the malignancy risk associated with the use of JAK inhibitors was limited in long-term data and seemed to be similar to those reported in biologic DMARDs (Curtis et al., 2016). Nevertheless, since most JAK inhibitors have been used for a short period after marketing, except for tofacitinib, and have a theoretical risk, it is necessary to review the occurrence of cancer-related AEs continuously and share the information in the drug label.

Recently, Peng et al. reported the results of a study analyzing the AEs of baricitinib using FAERS data and found that infections and hepatobiliary disorders were significant SOCs related to drug use (Peng et al., 2020). On the other hand, the potential SOCs of baricitinib found in this study were infections and surgical/medical procedures. We analyzed the AEs in which baricitinib was considered the primary suspected drugs unlike the study by Peng et al. (Peng et al., 2020). Hepatic enzyme elevation was also reported from both spontaneous reporting databases with an ROR of 3.04 (95% CI, 2.59–3.58). Since JAK inhibitors have a risk of liver transaminase increase, continuous monitoring is necessary for patients taking the drug (Winthrop, 2017).

The results of our study provide insight into potential safety issues that need to be evaluated in further studies. Drugs might cause renal injury by increasing serum creatinine levels (Winthrop, 2017). In addition, there have been a few cases of gastrointestinal perforation, and this study showed hematochezia as the potential IME associated with the use of tofacitinib, with an ROR of 2.44 (95% CI, 2.00–2.98). Although the correlation between JAK inhibitors and gastric perforation has not been clearly established, the online patient review in this study presented gastrointestinal disorders such as upper abdominal pain and dyspepsia. Therefore, close monitoring for gastrointestinal side effects is required in patients taking this drug (Winthrop, 2017). Although nephrolithiasis and cataracts were reported as potential IMEs associated with the use of JAK inhibitors, the causal relationship between JAK inhibitors and the occurrence of these AEs needs careful interpretation due to a high incidence of renal stones or cataracts in patients with RA (Oglesby et al., 1961; Ito et al., 1997).

Although this study showed a potentially insightful relationship between the use of JAK inhibitors and reporting of AEs in the real world using the spontaneous AE reports from the United States FDA and Korean KIDS as well as online patient reviews, it has several limitations. First, due to the nature of the spontaneous reporting database, it is uncertain whether the reported events were due to the suspected drug, underlying disease of the patient, or some other cause. Therefore, the primary suspect designation in the FAERS registry and the exception of reports with unlikely or not applicable causality in the KAERS registry were applied in the analysis. Second, since many cases are not reported spontaneously to related registries such as FAERS and KAERS, including duplicate and incomplete reports, we used a disproportionality analysis to find a statistical association between the drug and AEs, including ROR, PRR, and IC. Furthermore, we comprehensively analyzed the online patient review dataset as well as the spontaneous reporting database. Third, given the breadth of systems that prescription drugs can affect, some of the potential AEs analyzed here might be associated with the disease itself. Since the removal of disease-related AEs on a broad scale may mask instances where drugs lead to exacerbations of underlying disease, we only omitted the RA-related AEs from analysis, so it cannot be stated that our methods excluded all of them (Hoffman et al., 2016). Finally, the reporting rates may vary over time (i.e., higher post approval and diminishing over time or peaking after the addition of labeling warnings by the FDA), and most JAK inhibitors are new drugs with limited experience of post-market use. Therefore, further studies on the potential AEs of JAK inhibitors are warranted using additional real-world data, including analyses of administrative claims databases or electronic medical reports.

In conclusion, the present study suggested that the commonly reported potential AEs after the use of JAK inhibitors were increased risk of infection and thromboembolism. Unexpected AEs, such as malignancy and respiratory disorders, might also occur. However, there were some differences in the potential AEs frequently reported by JAK inhibitors. When an online patient review was integrated, ineffectiveness of the drug and gastrointestinal AEs were frequently reported for tofacitinib, infection was frequently reported with baricitinib, and symptoms related to pain or edema were mostly reported for upadacitinib. Since baricitinib and upadacitinib have been used for a relatively short period after marketing compared to tofacitinib, further research using various real-world databases is necessary to find potential AEs related to JAK inhibitors in patients with RA.

## Data Availability

The original contributions presented in the study are included in the article, further inquiries can be directed to the corresponding author.
